# Primary cilia utilize glycoprotein-dependent adhesion mechanisms to stabilize long-lasting cilia-cilia contacts

**DOI:** 10.1186/2046-2530-1-3

**Published:** 2012-04-25

**Authors:** Carolyn Ott, Natalie Elia, Suh Young Jeong, Christine Insinna, Prabuddha Sengupta, Jennifer Lippincott-Schwartz

**Affiliations:** 1 Cell Biology and Metabolism Program, National Institute of Child Health and Human Development, Bethesda, MD, USA; 2 Molecular Medicine Program, National Institute of Child Health and Human Development, Bethesda, MD, USA; 3 Department of Ophthalmology and Vision Science, University of California, Davis, CA, USA

**Keywords:** Primary cilia, Contact, Adhesion, Direct sensing, Glycoprotein

## Abstract

**Background:**

The central tenet of cilia function is sensing and transmitting information. The capacity to directly contact extracellular surfaces would empower primary cilia to probe the environment for information about the nature and location of nearby surfaces. It has been well established that flagella and other motile cilia perform diverse cellular functions through adhesion. We hypothesized that mammalian primary cilia also interact with the extracellular environment through direct physical contact.

**Methods:**

We identified cilia in rod photoreceptors and cholangiocytes in fixed mouse tissues and examined the structures that these cilia contact in vivo. We then utilized an MDCK cell culture model to characterize the nature of the contacts we observed.

**Results:**

In retina and liver tissue, we observed that cilia from nearby cells touch one another. Using MDCK cells, we found compelling evidence that these contacts are stable adhesions that form bridges between two cells, or networks between many cells. We examined the nature and duration of the cilia-cilia contacts and discovered primary cilia movements that facilitate cilia-cilia encounters. Stable adhesions form as the area of contact expands from a single point to a stretch of tightly bound, adjacent cilia membranes. The cilia-cilia contacts persisted for hours and were resistant to several harsh treatments such as proteases and DTT. Unlike many other cell adhesion mechanisms, calcium was not required for the formation or maintenance of cilia adhesion. However, swainsonine, which blocks maturation of N-linked glycoproteins, reduced contact formation. We propose that cellular control of adhesion maintenance is active because cilia adhesion did not prevent cell division; rather, contacts dissolved during mitosis as cilia were resorbed.

**Conclusions:**

The demonstration that mammalian primary cilia formed prolonged, direct, physical contacts supports a novel paradigm: that mammalian primary cilia detect features of the extracellular space, not just as passive antennae, but also through direct physical contact. We present a model for the cycle of glycoprotein-dependent contact formation, maintenance, and termination, and discuss the implications for potential physiological functions of cilia-cilia contacts.

## Background

Cilia extending away from a cell are ideally positioned to passively sample the extracellular environment. Primary cilia, motile cilia, and flagella are structurally and functionally similar microtubule-based organelles. Critical signaling compounds encounter receptors on cilia and influence cell function, homeostasis, and differentiation (reviewed in Veland *et al*. [1]). Because of their essential roles in receiving signals from the extracellular space and transmitting the signals into the cell, primary cilia are often described as cellular antennae [2].

Interestingly, several essential, contact-dependent functions of motile cilia have been described. Some unicellular organisms utilize flagellar adhesion during mating [3,4]. The best-studied example of flagellar adhesion occurs during mating of *Chlamydomonas reinhardtii*. Mating-type-specific glycoproteins, called agglutinins, on the surface of the flagella bind to the agglutinins of the opposite mating type [5]. This initiates a signaling cascade that triggers cellular changes required for cell fusion and zygote formation [6]. Motile cilia in the mammalian, female reproductive tract beat to generate fluid flow, but also function by binding to the oocyte cumulus complex to promote directed movement of the oocyte over the steep entrance of the infundibulum [7,8]. Marine larvae also use a combination of cilia movement to generate flow, and cilia adhesion to promote particle capture during feeding [9]. In the auditory system, cadherin has been shown to be critical to link stereocilia tips to kinocilia [10-12].

We hypothesized that like motile cilia, primary cilia have the potential to form adhesions. We examined cilia in two different tissues: photoreceptors in the retina and cholangiocytes in liver. In both of these environments we observed cilia form contacts with each other. Using a cell culture model we demonstrated that cilia from nearby cells could form persistent, regulated, glycoprotein-dependent, cilia-cilia adhesions. In addition, we found evidence for cellular control of adhesion release. We suggest that like the contacts made by motile cilia, adhesion of primary cilia is functionally relevant. These results also suggest that mammalian primary cilia may be more than passive, solitary receivers.

## Methods

### Transmission electron microscopy

Mice were euthanized by asphyxiation with CO_2_. Eyes were fixed by immersion in a solution of 2.5% glutaraldehyde, 2% paraformaldehyde in 0.1 M sodium cacodylate buffer, pH 7.4, and further dissected into eyecups for fixation overnight. Eyecups were post-fixed with 1% OsO_4_, and dehydrated through ethanol series. Eyecups were embedded in epon resin for ultrathin sectioning (100 nm) with a Leica EM UC6 ultramicrocotome (Leica Microsystems, Bannockburn, IL). Ultrathin sections were mounted on grids and stained with uranyl acetate (3.5% in 50% methanol) and lead citrate. Images of the sections were acquired with a transmission electron microscope (CM 120, Phillips Biotwin Lens, F.E.I. Company, Hillsboro, OR, USA) coupled to a digital camera (Gatan MegaScan, model 794/20, digital camera (2 K × 2 K), Pleasanton, CA) at the Diagnostic and Research Electron Microscopy Laboratory at UC Davis.

### Immunofluorescent analysis of liver sections

Normal C57BL/6 mice at postnatal day 2 and 1 year old were deeply anesthetized and sacrificed. Four percent paraformaldehyde in PBS was used to fix tissues through cardiac perfusion. Five micron liver sections were generated and treated with Xylene to remove paraffin. Antigen retrieval was performed with 0.01 N sodium citrate (pH 6.4) using a microwave with decreasing intensity. Sections were incubated with Endogenous Biotin Blocking Kit (Invitrogen 4303) following the manufacturer's protocol. Sections were blocked with normal goat serum (4%, Jackson Immunoresearch, 005-000-121), ovalbumin (2%, Sigma, A5503), and quenching antibody (goat anti-mouse IgG, 50 ug/mL, Jackson Immunoresearch, 115-005-003) for 4 h and incubated with mouse anti-acetylated tubulin (1:250, Invitrogen, 322700), or rabbit detyrosinated tubulin (1:250, Millipore AB3201) and mouse anti gamma tubulin (1:1000, Sigma, T6557) overnight. The next day, sections were incubated with biotin-conjugated goat anti-mouse secondary antibody (1:800, Jackson Immunoresearch, 115-065-146) for 1 h followed by Cy3-conjugated streptavidin (1:800, Jackson Immunoresearch, 016-160-084) or a cocktail of Alexa Fluor 546/488-labeled goat anti-mouse and rabbit IgGs. To detect nuclei, sections were incubated with Hoechst 33258 (2 mg/mL; Invitrogen) in PBS for 10 min, washed, then mounted for analysis.

### Cell culture and plasmids

MDCK cells were maintained in MEM with 10% FBS and 3 mM glutamine. Cell culture reagents were from Mediatech (Manassas, VA, USA) unless otherwise noted. Stable lines were selected and maintained in medium containing G418 (approximately 700 μg/mL). To promote increased polarization, 4 × 10^4 ^cells were seeded on a transwell filter (12 mm diameter, 0.4 μm pore, Corning) and grown for 6 to 9 days. We plated cells on the underside of the filter to generate inverted cultures for live cell imaging [13]. The method used to generate three-dimensional cultures has been described in detail [14]. Plasmids were provided by Yoshiuki Wakabayashi (tubulin) and Phil Beachy (SmoYFP). The YFP in SmoYFP was replaced with Cerulean by subcloning at unique AgeI and SalI sites. Populations of cells stably expressing the fluorescent chimeras were enriched by FACS sorting (NEI Flow Cytometry Core).

### Live cell imaging

Cells were transferred to CO_2 _independent media (Invitrogen, 18045088) and kept at 37°C. For imaging, the cells grown on the underside of the transwell were placed in 50-100 μL media in a LabTek chamber (Nunc) and covered to minimize effects of evaporation. To assay for contact disruption, we located cilia adhesions in cells that had been left in the imaging conditions for at least 4 h. Then, we added media to the apical side of the cells so the well could be lifted off the glass, carefully wicked the media from the side of the transwell, placed the transwell in the indicated solutions, re-located the identical cilia and acquired z stacks for at least 2 h. The assay using 2 mM EGTA was performed in a calcium-free Hanks Buffered saline solution (Mediatech, Manassas, VA). To assess contact formation, cells on 12 mm transwell filters were rinsed in CO_2 _independent media and filled with 400 μL on the basal side. The apical surface was placed in 50-100 μL CO_2 _independent media supplemented with 4 mM glutamine and indicated solution (2 μg/mL swainsonine, Sigma). The percentage of cilia making contact was quantified from z-stack images collected immediately after removal from the incubator, or after 12 h of treatment. As an alternative method of cilia contact quantification, we added DOPE rhodamine (Avanti polar lipids) to the cells immediately after removal from the growth conditions, stained twice for 5 min each in 50 μg/mL DOPE rhodamine, then rinsed twice quickly, and a third time for 5 min. To characterize contact formation and persistence, cells were set up the same way, and z stacks were collected every 4 to 5 min for 12 h (formation) or every 10 min for more than 60 h (persistence). To induce mitosis in polarized MDCK cells, we added 10 ng/mL hepatic growth factor (kind gift of Donald Bottaro) to the basal side of the cells as they were transferred to the imaging conditions. Confocal images were collected on a Zeiss LSM 510, LSM710, 3i Marinas, or Olympus Fluoview 1000 microscope system.

### Immunofluorescence

Cells were rinsed (1 × PBS) and fixed (4% parafomaldehyde, 1 × PBS) in 37°C solutions and kept at 37°C to preserve cilia structure. Staining was performed by the following sequence: 30 min block/permeabilization; primary antibody incubation overnight at 4°C, three 5-min rinses, secondary antibody for 30-60 min, three 5-min rinses. When needed, 2 μg/mL Hoechst 33258 (Invitrogen) was used to stain cells for 10 min prior to the final rinsing steps. For block, permeabilization and antibody incubation the following buffer was used: 0.1% saponin (Sigma), 1% BSA (Fraction V, Sigma), and 1 × PBS. The rinse buffer lacked saponin. Primary antibodies used included: mouse anti acetylated tubulin, 1:750 (Zymed, 32-2700; Invitrogen, 322700); rabbit detyrosinated tubulin, 1:500 (Millipore AB3201); ZO1, 1:100 (Invitrogen, 40-2200); mouse anti gamma tubulin, 1:5000 (Sigma, T6557); rabbit anti polycystin2 (H-280, Santa Cruz, sc-25749); and a rabbit antibody raised against recombinant GFP [15]. Secondary antibodies included: goat anti-mouse Cy3 (Jackson Immunoresearch, 115-165-146, 1:500, or 1:750), goat anti-rabbit Cy3 (Jackson Immunoresearch, 111-165-003 Alexa Fluor 488 goat anti-rabbit (Invitrogen, A11008, 1:500), goat anti-mouse Cy5 (Jackson Immunoresearch, 115-175-146), and goat anti-rabbit Cy5 (Jackson Immunoresearch, 111-175-003, 1:500).

### Scanning electron microscopy

Prior to plating cells, anopore transwells (Nunc) were coated with carbon or gold and sterilized by exposure to ultraviolet light for at least 1 h per side. Then cells were cultured and grown as described above. After growing for 7 days the cells were fixed in 4% paraformaldehyde, 1.5% gluteraldehyde for 10 min, then quenched for 30 min in 1 × PBS, 50 mM glycine. The following staining procedure was repeated twice: cells were rinsed three times in 0.1 M Hepes or water, stained in 1% osmium tetroxide in the dark for 20 min, rinsed three times in water and stained for 20 min in 1% tannic acid. All SEM reagents were from Electron Microscopy Sciences, Hatfield, PA. The samples were dehydrated and critical point dried prior to imaging on a Hitachi 4800 SEM.

### Image analysis and processing

Images were collected using the proprietary software of each instrument listed above. Image analysis was also performed using Image J. Projection images were created using Volocity (Perkin Elmer) or Zen (Zeiss).

## Results

### Evidence and characterization of cilia contact

**Figure 1:**
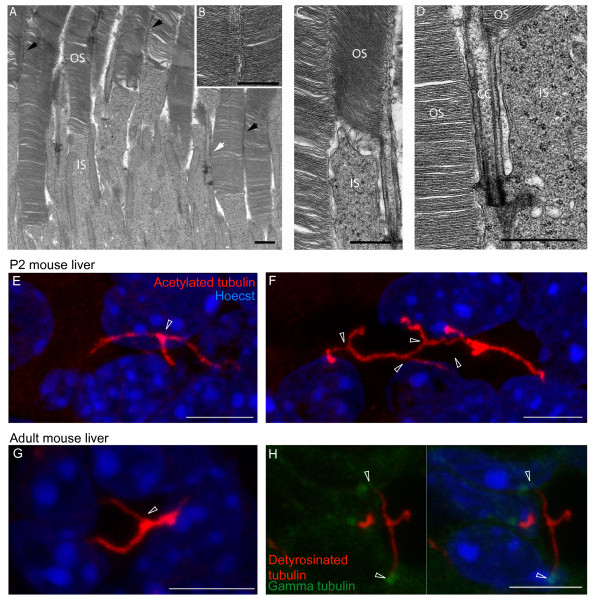
**Mammalian ciliary membranes form direct contacts in vivo**. (**A-D**) Electron micrograph views of photoreceptor inner segments (IS) and sensory cilia (OS) in a mouse retina. Close apposition of immediately adjacent OS membranes is visible (black arrows in **A**, **B**, **C**). OS membrane of a rod cell forms contact with the connecting cilium (CC) of a neighboring cell (white arrow in **A**, **D**). (**E-H**) Cilia from different cholangiocytes come together. Images are maximum intensity projections from confocal z-stacks of immunostained, paraffin-embedded mouse liver sections from P2 (**E **and **F**) and adult (**G **and **H**) mice. White arrowheads in **E**, **F**, and **G **indicate cilia contact; in H the arrowheads are at the centrosomes. Hoechst staining shows the position of nuclei (blue); acetylated (**E**-**G**) or detyrosinated (**H**) tubulin staining was used to visualize primary cilia (red); and gamma tubulin staining indicates the base of the cilia in H. Scale bars are 1 μm in **A**-**D **and 5 μm in **E**-**H**.

Primary cilia have the right geography for sensing the extracellular environment. To test our hypothesis that primary cilia physically interact with their surroundings, we looked in two different cilia environments: the retina and the liver. In the retina, photoreceptor cells extend a modified cilium. The ciliary membrane of rod cells encapsulates both the connecting cilium, which is a conventional axoneme, and the outer segment, which includes an ordered stack of opsin-rich disk membranes [16]. Rod cells and their cilia are packed tightly together within the photoreceptor layer. We examined transmission electron micrographs of sections through mouse retinas and observed potential adhesion sites between the ciliary membranes of rod outer segments and directly adjacent outer segments (Figure 1A, B, C). In addition, the membrane of connecting cilia also appeared to have extended regions of contact with neighboring outer segments (Figure 1A, D). The close proximity of the two membranes resembles seams that bind the outer segment layer together.

Cholangiocyte cilia protrude into the lumen of the bile ducts and have been shown to sense changes in tonicity, flow speed and signaling molecules [17]. To see what cilia in a lumen might contact, we examined paraffin embedded mouse liver sections from both 2-day-old (P2) and 1-year-old mice. Most cilia in the large ducts of the adult mice did not contact anything; however, in the smaller ducts cilia from nearby cells occasionally contacted one another (Figure 1G, H). We also observed cilia from many cells come together and form a network of cilia (Figure 1F). In the younger animals the diameter of the duct is smaller and cilia are often close together. We found many more examples of P2 cholangiocyte cilia contacting one another (Figure 1E, F).

**Figure 2:**
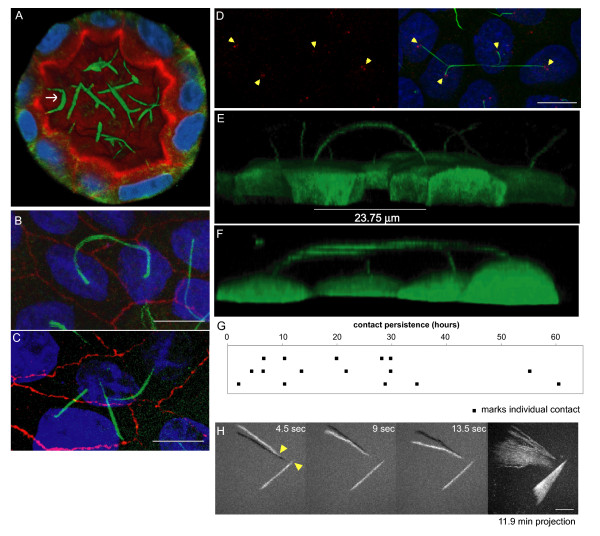
**Prolonged cilia contacts form in cultured cells**. (**A**) MDCK cells were cultured in a matrix to form a three-dimensional cyst, then fixed and stained. The arrow indicates an area of overlapping contact (acetylated tubulin - green; Hoechst - blue; actin - red). (**B**, **C**) MDCK cells were grown on transwell filters, fixed, stained, and imaged using a confocal microscope. These maximum intensity projection images show overlapping (**B**) and point (**C**) contacts between cilia (acetylated tubulin - green; Hoechst - blue; ZO1 - red). (**D) **Filter grown MDCK cells were stained and imaged on a confocal microscope (Hoechst - blue, anti-detyrosinated tubulin - green, and anti-gamma tubulin - red). The image presented is a maximum intensity projection (the contrast in the red channel was changed to make the centrosomes more visible). (**E**, **F**) Filter grown MDCK cells stably expressing tubulin-GFP were imaged by confocal microscopy and used to generate projection images. Cilia from nearby cells appear to be coming together. (**G**) We monitored cells during contact formation and measured how long cilia remained together to determine whether the contacts are transient chance encounters or more persistent inter-cellular interactions. Each point on the graph represents a contact event. The shortest contact duration observed was just over 2 h. (**H**) To test whether primary cilia can move we collected rapid z stacks of primary cilia from TubGFP expressing cells at 4.5 s intervals using a spinning disk confocal microscope. We generated a time series of the maximum intensity projections of the z stacks and then subtracted the preceding frame from each image; thus, the shadow indicates the position of the cilium at the previous time point. Yellow arrowheads in the first panel indicate the base of each cilium. The end panel is a projection of the position of the cilia at every time point for 11.9 min (scale bar is 10 μm in **B**, **C**, and **D**; 5 μm in **G**).

When seeded in collagen, Madin-Darby canine kidney (MDCK) epithelial cells form three-dimensional cysts with cilia on the apical surface oriented to the lumen. We examined cilia in this cultured cell system to determine whether these cilia form connections. Imaging both fixed and live cysts, we found evidence that cilia in the lumen form contacts similar to those observed in the two-dimensional culture (Figure 2A and data not shown).

Taken together, the in vivo and three-dimensional culture data support the hypothesis that cilia can form direct contacts; specifically, they contact one another. These cilia-cilia contacts resemble the flagella-flagella contacts characterized in *Chlamydomonas *[6]. Previous reports have described possible cilia touching in prenatal pancreatic tissue and cultured cells [18,19], but no previous work has defined or characterized these observations. To study cilia-cilia contacts in a quantitative manner we examined the cilia of tightly packed, filter-grown Madin-Darby canine kidney epithelial cells (MDCK cells), which, after 7 days of growth, have cell diameters of approximately 10 μm and cilia lengths ranging from 8 to 15 μm, occasionally longer. Under these growth conditions, we observed primary cilia that appeared to touch each another.

In some cells cilia appeared to overlap: cilia extended from the central region of the apical surface of each cell, rose away from the cell body and joined together to form a continuous arc (Figure 2B). In other cases, the tip of one cilium touched the shaft of a cilium from a different cell forming a point contact (Figure 2C). Contact between cilia was observed in both fixed, immunostained cells (Figure 2B, C, D) and living cells expressing fluorescent proteins like tubulin-GFP (Figure 2E, F, and Additional file 1: Movie S1 and Additional file 2: Movie S2). Three-dimensional projections such as the one shown in Figure 2F (Additional file 2: Movie S2) revealed that in addition to connections between two cilia, cilia also form networks, where the cilia from three or more cells come together. Other cell types examined, including NIH3T3, IMCD-3, and C3H/10 T1/2 cells, did not form contacts, probably because cilia in most cultured cells are shorter and cannot reach each other.

To determine whether the physical contacts were transient chance encounters or persistent cell-cell adhesions, we assayed the duration of newly-formed cilia adhesions. We set cells in 100 μL of CO_2_-independent media and recorded z stacks over time as cilia contacts formed. We measured the duration of the contacts. Once established, cilia contacts persisted for at least 2 h, and half continued more than 20 h. A few even lasted for more than 2 days (Figure 2G and Additional file 3: Movie S3). These data indicate that the physical contacts made by primary cilia are not transient, but rather are quite stable.

Primary cilia are often termed non-motile because their axoneme generally lacks the dynein proteins on the outer and inner microtubule doublets of the axoneme that are responsible for cilia beating. However, we realized that some type of primary cilia movement should promote cilia-cilia encounters in a lumen or in the MDCK culture system. To test whether primary cilia move, we recorded cilia position over time. As shown in the projection series of Figure 2H and Additional file 4: Movie S4, the position of cilia can shift over time. This movement can be gradual (lower cilium), or more rapid and sporadic (upper cilium). The ciliary movement does not appear to involve ciliary bending and thus may be distinct from the well characterized beating of convention motile cilia. Nearby cilia do not move in unison as would be expected if their movement was caused by currents. Shifting of cell or centrosome position may contribute to cilia movement. Indeed, our data demonstrate that the base of one cilium moves during the course of the time series. Additional mechanisms may also contribute to the movement of primary cilia. Both sporadic and gradual movements could facilitate cilia-cilia contacts by bringing cilia in proximity of one another.

We used two methods to quantify cilia contact in live MDCK cultures. First, we assessed the number of cilia making contact in cells expressing tubulin-GFP immediately after transfer to imaging media. We found that 13% of cilia made contact (*n *= 687; standard error = 2%). Our stable tubulin-GFP cell line contains a mixed population of GFP positive and negative cells. Although we restricted our analysis of cilia contacts to fields with high density of GFP positive cells, some connections may have been missed. As an alternative method, we stained the apical surface of live cells with the lipid dye DOPE rhodamine. Although the lipid moves away from the cilium over time, fluorescence is visible for at least 30 min, which is long enough to collect z stacks of several fields of view. In this assay we also measured 13% (*n *= 566 cilia; standard error 1.9%) of cilia making contact.

### Models for cilia-cilia contact

**Figure 3:**
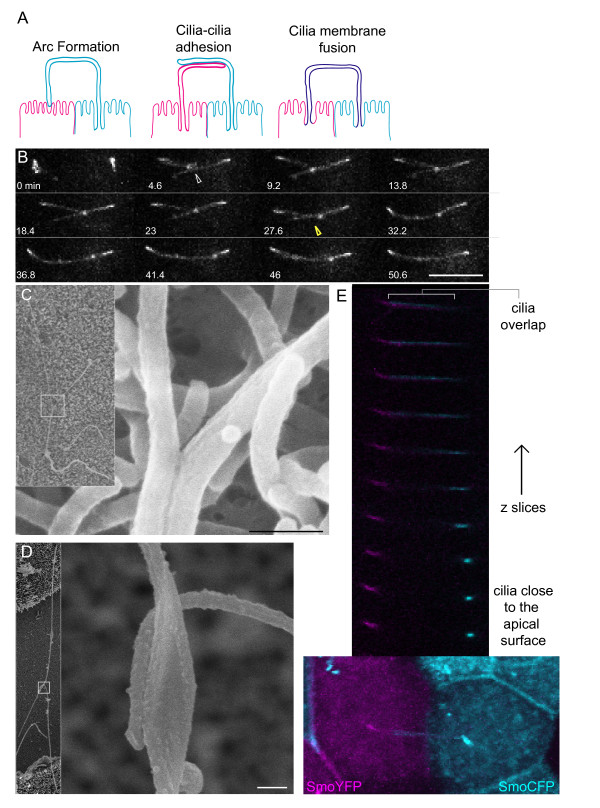
**Contact results in adhesions of cilia membranes**. (**A**) Multiple models could explain the observed coincidence of signal from multiple cilia: one cilium could be resorbed and the other could be making contact with the apical surface, or the membranes of cilia could adhere to one another, or the cilia membranes could be fusing to create an intercellular connection. (**B**) The formation of the cilia adhesion was monitored by collecting z stacks every 4.6 min. This montage shows the maximum intensity projections of the cilia of two adjacent cells during contact initiation. It appears that a point contact is made (white arrowhead) and maintained for a short time before the area of adhesion starts to spread (yellow arrowhead). Scale bar is 10 μm. (**C**, **D**) Scanning EM images of cilia engaged in overlapping (**C**) and point (**D**) adhesions (scale bar is 0.2 μm). (**E**) Pools of cells stably expressing SmoYFP and SmoCFP were mixed and plated on the same transwell. The lowest panel is a maximum intensity projection image of cilia adhesions between cells expressing different fluorophores. Individual z slices make up the montage above and demonstrate that the cilia remain distinct: fluorescence is not shared between the two adjacent cilia.

Several models could account for our observation of apparent cilia contact: (1) A single cilium could be bent into an arc; (2) individual cilia could adhere to one another; or (3) the primary cilia from different cells could fuse (see diagrams in Figure 3A). To differentiate between these models, we used both confocal imaging and scanning electron microscopy (SEM). In the first approach, we investigated the process by which cilia come together. We collected confocal z stacks over time. As shown in Figure 3B, cilia contact appeared to be initiated by a tip to shaft contact. Over the course of 30 to 60 min, the contact area extended until all overlapping membrane surfaces were engaged in contact. These results combined with our earlier observation that cilia from multiple cells seem to come together, ruled out the possibility that one cell extends a long cilium. Finally, these results also indicated that contact formation involves a two-step process: contact initiation and adhesion progression.

To determine whether cilia membranes fuse after coming together we examined sites of cilia contact using SEM. Figure 3D shows an example of a point contact. Interestingly, the plasma membrane of one of the cilia appeared expanded at the site of contact. A few cells with overlapping adhesions had twisted cilia, however, in most cases, individual cilia were distinct and adjacent. We did not detect any trace of particles between individual cilia when the contact point was imaged at high-magnification (100,000× and 200,000×).

We used confocal microscopy to look for fusion of membranes or exchange of membrane proteins between cilia. We plated a mixture of cells stably expressing either SmoYFP or SmoCFP on the same transwell, and then searched for contact between cilia of cells expressing different fluorophores. An example is shown in the z-stack montage in Figure 3E (see also Additional file 5: Movie S5). In every case, individual cilia were discernable. The fluorescent membrane proteins did not mix or co-localize indicating that the ciliary membrane composition remains distinct and the membrane proteins did not exchange between cilia. In addition, a region of overlap could be seen in z slices 1 to 3. These results argued against models of bent cilia or cilia fusion. Taken together, the confocal and SEM data indicate that the ciliary membranes are not fusing, but rather cilia form adhesions through direct contact.

### Glycoprotein-dependent adhesion mechanism

**Figure 4:**
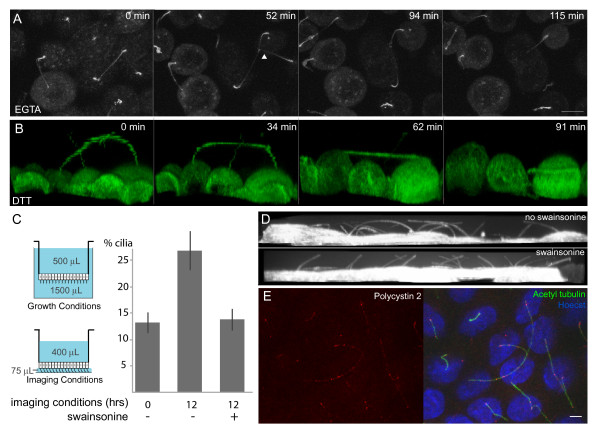
**Cilia-cilia adhesions are glycoprotein-dependent, but not calcium- or disulfide- dependent**. (**A**) Many adhesion proteins including integrins and cadherins require calcium. To assess the calcium requirement of the inter-cilia adhesion, cilia making contact were located, then the sample was moved to a solution of 2 mM EGTA, the same field of cells was re-located, and the cilia adhesion was monitored over time. The maximum intensity projections show that the adhesion between the cilia persisted long after the cadherin-based adhesions have been disrupted (cells round up) and adhesion of an additional cilium occurred in the absence of calcium (arrowhead in second panel). (**B**) Adhesion molecules containing immunoglobulin-like domains require disulfide bonds. We monitored cilia contact after addition of 10 mM DTT. The XZ projection image shows that the treatment causes the cells to deform and come off the filter, yet the cilia adhesion persists for more than 1 h. (**C**) We observed that leaving cells in the imaging conditions for 12 h resulted in an increase in the formation of contact relative to cells directly removed from the growth conditions (*P *value < 0.0005). We took advantage of this observation to assay the ability of the mannosidase II inhibitor, swainsonine, to prevent stable cilia contacts. We found that in the presence of 2 μg/mL swainsonine the number of cilia contacts was similar to cells directly removed from the growth media, indicating that mature glycoproteins promote the formation of cilia contacts (*P *< 0.005). Each value is calculated from more than 640 cilia in three different samples. (**D**) Side projections of cells in the imaging conditions for 12 h without (upper) or with (lower) swainsonine treatment. (**E**) During flagellar adhesion in *Chlamydomonas reinhardtii *the cilia localization of polycystin 2 increases. We used an antibody to polycystin 2 to demonstrate that polycystin 2 localization is similar in cilia that adhere to other cilia and cilia that are free. Scale bars are 5 μm.

To understand the nature of the cilia-cilia adhesion, we attempted to dissolve the physical attachments between cilia. To assay acute treatment effectiveness we located cilia contacts, moved the transwell to a treatment solution, located the same cells again, and monitored each cilia-cilia contact for up to 2 h (the minimum persistence time reported in Figure 2F) or until the integrity of the monolayer was significantly disturbed. We hypothesized that integrins or cadherin-like molecules mediated the cilia adhesion. Previously, Praetorius *et al*. reported that α3, α5, and β1 integrins are present on MDCK primary cilia [20]. Cadherins also seemed like a good candidate because the linkages between kinocilia and stereocilia are mediated by these proteins [11,12]. Both integrins and cadherin-like proteins are calcium-dependent [21,22], so to test for their involvement we chelated calcium with 2 mM EGTA. As shown in Figure 4A, we observed disruption of the cadherin mediated, cell-cell, tight junction adhesions without any effect on cilia-cilia contacts. We were also able to record initiation of cilia contact in the absence of calcium (also see Additional file 6: Movie S6). These results indicate that calcium is not required for initiation or maintenance of cilia contacts. Therefore, neither integrin nor cadherin-related adhesion molecules were responsible for the contacts between cilia. We saw similar results when we transferred cells to a solution containing 0.05% trypsin and 0.53 mM EDTA in Hank's buffered saline solution, which suggested that if proteins were involved in cilia contacts, they were either inaccessible to the protease or did not have the appropriate cleavage motif (Additional file 7: Movie S7).

Proteins containing Ig-like domains mediate many tight cell-cell adhesions. Polycystin1 is an important cilia protein whose large extracellular domain contains sixteen Ig domain-like repeats [23]. Adhesion molecules with Ig domains utilize disulfide bonding to maintain structure. To test the involvement of Ig-related adhesion molecules, we assayed cilia contact persistence in the presence of the reducing agent DTT. We observed maintenance of cilia-cilia adhesion long after the treatment significantly altered the cell morphology (Figure 4B and Additional file 8: Movie S8). Before the cilia localization of PKD1 was identified, it was hypothesized that homophilic interactions of Ig-like domains of PKD1 may play an important role in cellular adhesion at cellular junctions. This was supported by evidence that an antibody against the extracellular Ig-like domains of PKD1 perturbs cell contacts in subconfluent MDCK cultures [24]. We assayed the persistence of cilia contacts in the presence of the same anti-IgPKD antibody and found no effect. Taken together our data suggest that neither PKD1 nor other Ig domain containing proteins mediated the physical interactions between cilia.

Physical association of *Chlamydomonas *flagella initiates the mating process. Cells of opposite mating types express complimentary agglutinin glycoproteins that bind and initiate flagellar adhesion [25,26]. Interestingly, we observed an increase in the number of cilia contacts after imaging cells for 12 h in the contact formation and contact persistence assays. As shown in Figure 4C, the imaging conditions differ from the growth conditions in the volumes of media on the apical and basal sides of the membrane and the proximity of the cells/cilia to the bottom of the dish. We took advantage of this increase in contact to develop an assay to test the involvement of glycoproteins in contact formation. Specifically, we used swainsonine to inhibit mannosidase II, an enzyme that processes N-linked glycans in an early step of complex oligosaccharide synthesis [27,28]. To be sure that the swainsonine was active, we compared concanavalin A-rhodamine binding in treated and untreated cells. Because concanavalin A binds tightly to mannosyl groups that are removed by mannosidase II, we saw an increase in concanavalin A binding upon swainsonine treatment. In contrast, we observed no change in wheat germ agglutanin-rhodamine staining, which binds sialic acids. We did not assay the effect of acute swainsonine treatment because we reasoned that in the 2-h timeframe of the acute assay, only a small fraction of the cilia glycoproteins would be replaced by the immature glycoproteins generated upon swainsonine treatment. Instead we quantified the percent of cilia making contact after 12 h in the presence or absence of 2 μg/mL swainsonine when cilia would have used the immature glycoproteins to potentially form new contacts. As shown in Figure 4C and 4D, swainsonine prevented the two-fold increase in cilia contact observed in untreated cells. This result suggests that like flagellar adhesions in *Chlamydomonas*, mammalian cilia adhesions employ complex glycoproteins in either their formation or their maintenance.

We also tested the involvement of glycoproteins in cilia adhesion by assaying cilia persistence in the presence of neuraminidase (which removes sialic acid), elastase (which cleaves galectins [29]), and by swamping in free glucose; however, none of these perturbations disrupted cilia-cilia contacts (data not shown). It is possible that we were not able to disrupt established contacts because carbohydrates that we did not test are essential. Alternatively, multiple protein complexes could be involved, or access to adhesion molecules could be obstructed.

Upon flagellar adhesion in Chlamydomonas, a signaling cascade is initiated that involves flagellar localization of PKD2, calcium influx, and kinase activation [30,31]. We tested the possibility that similar pathways are involved in mammalian cilia adhesion. The image shown in Figure 4E is an example showing that, unlike Chlamydomonas flagellar adhesion, PKD2 localization in the cilium is not increased upon cilia contact formation. However, because these cells are fixed, we would not have detected transient changes in this assay.

### Cilia contacts melt prior to mitosis

**Figure 5:**
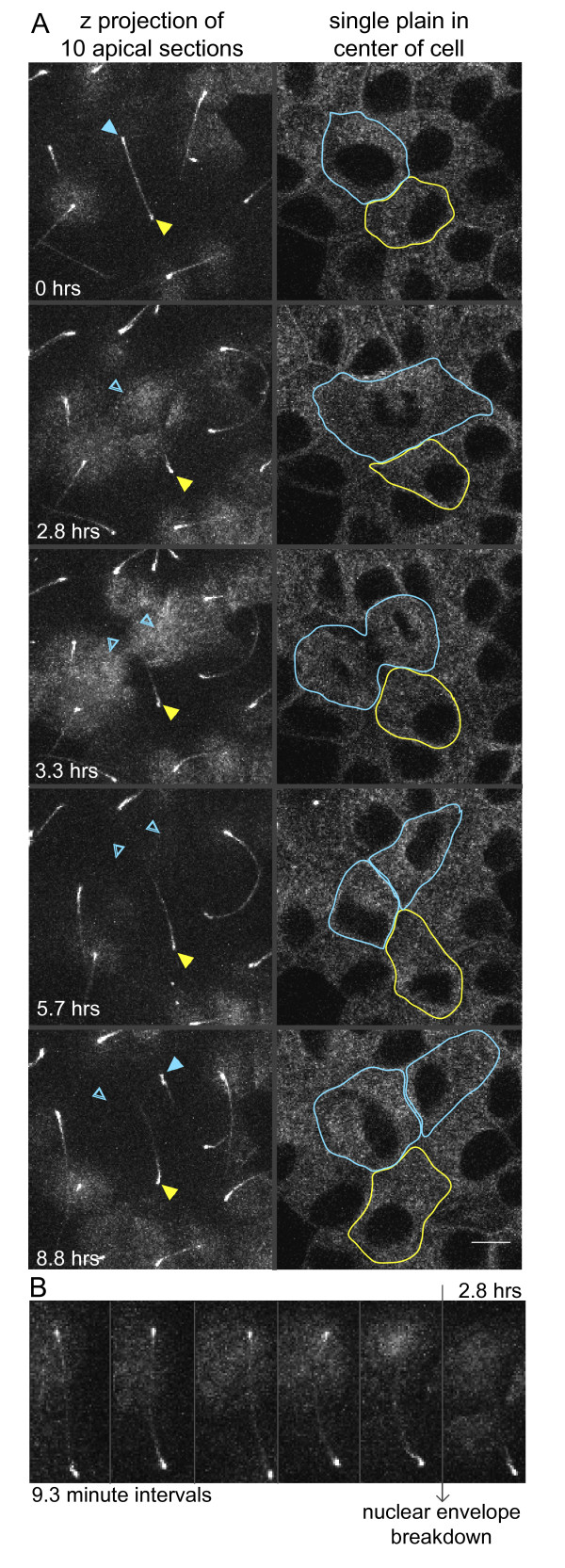
**Disassembly of cilia adhesion prior to cell division**. MDCK cells stably expressing SmoYFP and engaged in cilia contacts were treated with hepatic growth factor to induce cell division and cell movements. (**A**) Z-stack images were recorded every 9.3 min. The panels on the left are the maximum projection of z slices 1-10 (the apical surface of the cell). The filled arrowheads indicate the base of the cilia; the empty arrowheads indicate absent cilia. The right panel is a single section through the nuclei of the cell layer, where the progress of cell division is clearly visible. While the images of these cells were being collected, the upper cell (cyan) resorbed its cilium and divided. In the last frame, the cilium of one of the new daughter cells is visible. (**B**) Montage of the maximum projection of z slices 1-10 showing the five time points that directly proceed nuclear envelop breakdown. The loss of the upper cilium is visible. Brightness and contrast were adjusted to make cilia more obvious.

As indicated in Figure 2F, cilia do not stay attached indefinitely. This suggested to us that a regulated mechanism might dissolve the inter-ciliary adhesion. We examined a physiologically relevant situation where separating cilia contacts would be advantageous. Prior to mitosis mammalian cilia are resorbed [32]. We stimulated mitosis in polarized MDCK cells by adding hepatic growth factor [33] and monitored the status of cilia contacts. As shown in Figure 5 and Figure Additional file 9: Movie S9, we found that cells engaged in cilia-cilia contact dissolved the adhesion as they resorb. After mitosis, when cilia regrow, some of the cilia of daughter cells engaged in cilia contact with the original partner cell, with each other, or with new partner cells. The directed dissolution of cilia contact supported the hypothesis that the maintenance of physical contacts between cilia is a regulated cellular adhesion.

## Discussion

Many electron micrographs have shown that rod outer segments pack tightly. To our knowledge, this is the first report to suggest that the adjacent cellular membranes actually form stable adhesions. The possibility that these are bona fide cellular adhesions is supported by the observation that cilia in bile duct and in cultured kidney cells are also capable of forming and maintaining cilia-cilia contacts. We speculate that cilia in many other contexts may form contacts. For example, the cilia of amphidial neurons in *C. elegans *are packed very tightly and have ciliary membranes that are directly adjacent [34].

The studies performed here revealed that glycoproteins contribute to cilia-cilia adhesion. In addition, we provide evidence that other classes of adhesion molecules (cadherins, integrins, and immunoglobulin fold containing proteins) are not essential for contact maintenance. There is a precedent for the involvement of glycoproteins in adhesive cilia functions in other contexts. For example, the sugars on the mating type specific agglutinin proteins involved in *Chlamydomonas *flagellar adhesion are glycoproteins [5,26,35]. In addition, bacteria that bind to motile cilia membranes in the respiratory tract specifically recognize glycolipids on the cilia surfaces [36-38].

**Figure 6:**
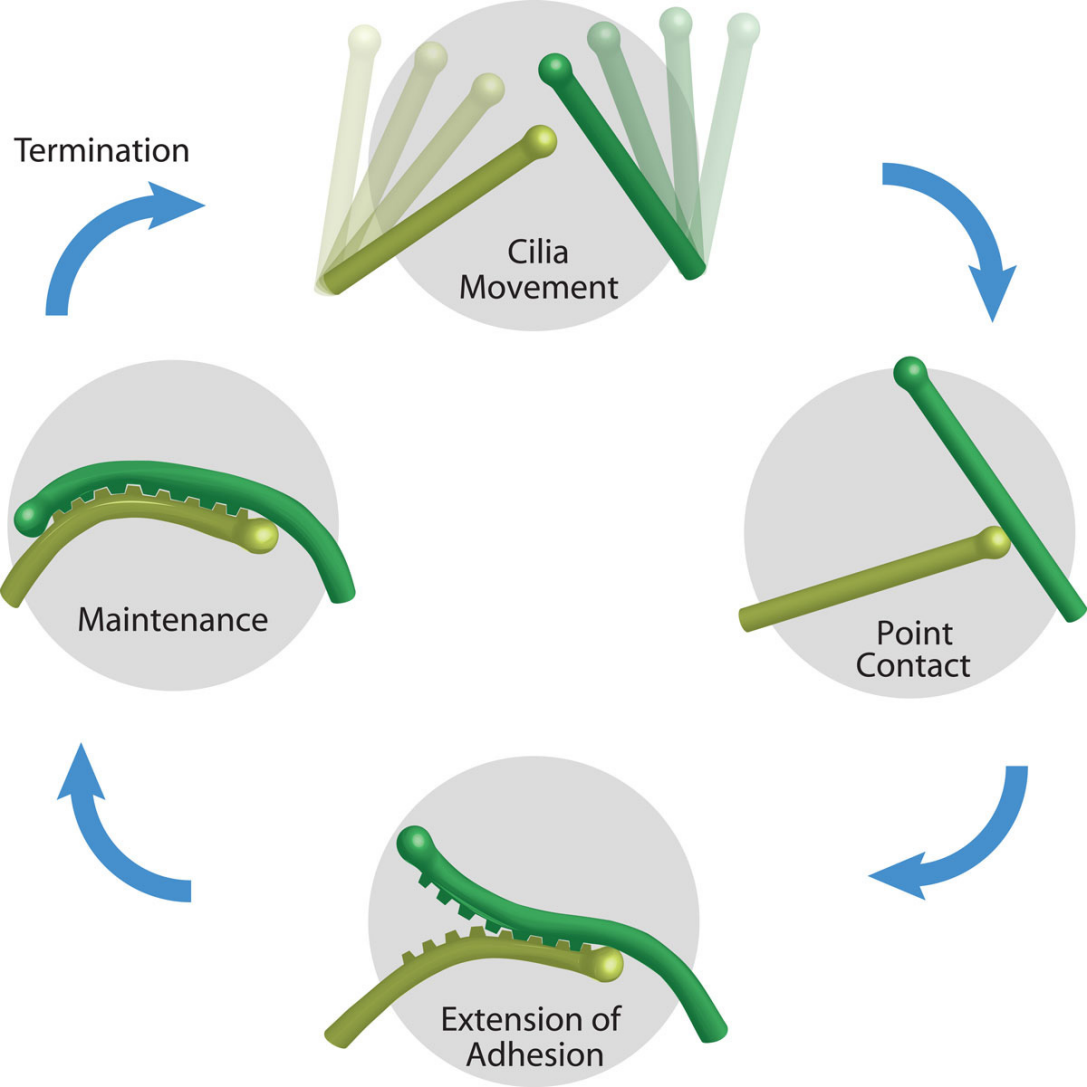
**The cilia contact cycle**. We propose a cycle of cilia contact formation, maintenance, and termination. Movement of primary cilia enables the tip of one cilium to contact the shaft of a nearby cilium. If adhesion molecules are engaged, then a point contact is formed. A zipper-like extension of the region of overlap creates a long-lasting, overlapping, cilia-cilia adhesion. By similarity to *Chlamydomonas *flagellar adhesion, we speculate that the maintenance of contact is an active process. When the contact is terminated, cilia are again free to move and may encounter another cilium.

Based on the data presented here, we propose a cycle of cilia adhesion formation, maintenance, and termination (Figure 6). The data in Figure 3B suggest that stable adhesion formation in MDCK cells is a two-step process. First, cilia movement leads to a point contact initiated by adhesion of one cilium tip to the shaft of an adjacent cilium. The cilia in Figures 2B and 3E are at this stage of contact. When quantifying cilia contact persistence we noticed (but did not include in our count) cilia that formed point contacts for short periods of time and then separated. The second step which is required to form overlapping adhesions, is stabilization of the point contact and adhesion extension to zipper the membranes together. Both point and overlapping contacts reduce the movement of primary cilia.

Once established, adhesions must be maintained. Protease treatments failed to disrupt established cilia-cilia adhesions. One explanation for this could be that multiple types of adhesive interactions contribute to cilia-cilia adhesion. Perhaps glycolipid interactions supported established contacts in the presence of trypsin. Alternatively, the proteases could have been excluded from the extended areas of overlap. Although we were not able to artificially disrupt cilia adhesions, cells can and do terminate cilia contacts. The ability of cells to dissolve adhesions immediately prior to dividing suggests that contact termination is a regulated process. Adhesion could break down due to turnover of adhesion molecules. Protein turnover and new protein synthesis has been shown to be important for maintenance of adhesion in cell fusion deficient *Chlamydomonas *mutants [39]. Glycosidases could also be deployed to disrupt cilia adhesion. Once liberated, primary cilia again are free to move and possibly encounter other cilia.

Cells could employ cilia-cilia adhesion for multiple purposes. It has been well established that many passively received signaling pathways initiate on ciliary membranes (such as the hedgehog pathway [40]). Cilia-cilia adhesion could create a platform that also initiates and perpetuates intracellular signaling. This seems plausible in light of the characterized signaling cascade mediated by *Chlamydomonas *flagellar agglutinin proteins during adhesion, as well as the many established signal-transduction cascades mediated by other types of adhesion proteins. Another possibility is that cilia adhesion forces two cilia to share a very similar space, which could increase the likelihood that nearby cells will have similar responses to external stimuli. We did compare the status of several signaling pathways in free and adhering cilia, but have not yet identified downstream consequences of cilia contact.

Strong adhesions could also restrict movements of cells within the monolayer. We tested this in the cell culture model and found no difference between the movements of cells with connected versus independent cilia when cell movement was stimulated by the addition of hepatic growth factor. We did find that cilia adhesion increased when the space on the apical surface decreased: more contacts were present in the smaller ducts of P2 mice, in the smaller MDCK cysts, and when the MDCK monolayer was left in the imaging conditions for long periods of time. One interpretation of this data might be that cilia adhesion could be used to monitor the size of a lumen.

An additional possibility is that stable cilia-cilia interactions provide structural support. Increased numbers of contacts in cilia networks restricts the movement and bending of cilia. We did assay the calcium flow response of cells engaged in cilia contact and found that the response of these cells was similar to cells with free cilia (similar to [41]). However, structural support could be important in tissues that are not exposed to flow- such as rod outer segments. We hypothesize that cilia-cilia adhesion may help maintain and support the retina architecture.

## Conclusions

Adhesion between cilia occurred in the retina, the bile duct, and in cultured cells. These contacts were not momentary kisses, but rather persisted for hours or days. The mechanism of adhesion was glycoprotein-dependent and resistant to several chemical manipulations. We observed that cilia adhesion disruption coincides with cilia resorption. Because the cilia contacts dissolve prior to mitosis, yet they are potentially long-lasting, we propose that maintenance of mammalian primary cilia adhesion is a regulated process.

The paradigm of contact-mediated cilia function may extend beyond cilia-cilia adhesion. Primary cilia function in many diverse cellular environments. Adhesion to other cells could also be important for cilia function in these contexts. Future studies exploring the contact of primary cilia to other cilia and other cells will likely reveal novel primary cilia functions that are relevant to both development and disease.

## Abbreviations

DOPE: Dioleoylphosphatidylethanolamine; DTT: Dithiothreitol; EDTA: Ethylenediaminetetraacetic acid; EGTA: Ethylene glycol tetraacetic acid; GFP: Green fluorescent protein; IS: Inner segment; MDCK: Madin Darby canine kidney; MEM: Minimum Essential Medium Eagle; OS: Outer segment; P2: Postnatal day 2; PBS: Phosphate buffered saline; SEM: Scanning electron microscope.

## Competing interests

The authors declare that they have no competing interests.

## Authors' contributions

NE cultured and imaged the 3D MDCK cysts. CI prepared and imaged the cilia in retina. SYJ prepared and stained liver sections. PS helped prepare and image SEM samples. CO performed all other experiments, analyzed the data, and wrote the manuscript. Both CO and JLS conceived and designed the experiments. All authors read and approved the final manuscript.

## Supplementary Material

Additional file 1 **Movie S1**. Cilia from live MDCK cells form overlapping contacts. The rotating projection of live TubGFP expressing MDCK cells shows that cilia from nearby cells made contact. There were two pairs forming contacts in this field of view, one of which is between a pair of cells that are not directly next to one another. Click here for file

Additional file 2 **Movie S2**. Cilia from live MDCK cells form networks of connections between multiple cells. The rotating projection of live TubGFP expressing MDCK cells shows that cilia from multiple cells adhere to one another and form a network of connected cilia. Click here for file

Additional file 3 **Movie S3**. Cilia contacts persist for many hours. This is a representative time series of maximum projection images of SmoYFP expressing cells created from z stacks that were acquired every 13 min for 65 h. During this time, contacts form between several cilia in the field of view. Cilia contacts were not transient. Rather, contacts lasted for hours or days. This is representative of the data used to quantify cilia contact shown in Figure 2F. Toward the very end of the series, cells are not as healthy. Click here for file

Additional file 4 **Movie S4**. Mammalian primary cilia movement. Maximum intensity projection images were generated from Z-stack images of TubGFP expressing MDCK cells collected every 4.5 s. To generate this movie the preceding image was subtracted from every time point (after background subtraction), leaving the appearance of a shadow when the cilia moved. Click here for file

Additional file 5 **Movie S5**. Cilia form overlapping adhesions. The cilium of a cell expressing SmoYFP (magenta) is adhering to the cilium of a cell expressing SmoCFP (cyan). This series is a z stack that starts at the cilia on the apical side of the cell and moves in the basal direction. The cilia from the two cells are adjacent. They do not fuse and there does not appear to be exchange of fluorescent membrane proteins between the cilia. Click here for file

Additional file 6 **Movie S6**. Calcium chelation failed to disrupt cilia contacts. EGTA was added to chelate calcium ions. The effect on cadherin-mediated adhesions between cells was obvious because the cells rounded up quickly. In contrast, the cilia-cilia adhesions were maintained for more than an hour. Interestingly, an additional cilium joined the adhesion, suggesting that neither formation nor maintenance were calcium-dependent. Click here for file

Additional file 7 **Movie S7**. Cilia contacts persist in the presence of Trypsin and EDTA. After locating cilia-cilia adhesions, cells expressing SmoYFP were transferred to a treatment solution that contained 0.05% trypsin and 0.53 mM EDTA in Hank's buffered saline solution. After a short time, the cells rounded up and started moving away from the filter. The cilia-cilia adhesions persisted until the integrity of the monolayer was significantly disturbed. Click here for file

Additional file 8 **Movie S8**. Maintenance of cilia-cilia adhesions did not depend on disulfide bonds. DTT was added to disrupt disulfide bonds. The effect on the monolayer is evident because the cells rounded up and shift positions. In spite of the DTT, cilia contacts persisted for more than 1 h. Click here for file

Additional file 9 **Movie S9**. Disassembly of cilia adhesion prior to cell division. MDCK cells expressing SmoYFP were treated with hepatic growth factor, which stimulated mitosis. Cilia contact did not prevent cells from entering mitosis. Rather, disruption of the cilia contact coincided with the cilium resoprtion of the dividing cells. Toward the end of the time series the cilia from each of the daughter cells become visible. Click here for file
